# Development of a protocol for intravenous infusion management in patients with aggressive behavior due to mental disorders: A Donabedian framework approach

**DOI:** 10.1371/journal.pone.0326717

**Published:** 2026-05-21

**Authors:** Liying Liu, Yaling Sun, Yujie Hao, Wanyu Zhang, Yan Gao, Yulian Wei, Lina Wang, Honghui Wang

**Affiliations:** 1 Hebei Provincial Mental Health Center, The Sixth Clinical Medical College of Hebei University, Baoding, China; 2 School of Nursing, Nanjing University of Chinese Medicine, Nanjing, China; 3 Huai’an No.3 People’s Hospital, Huaian Second Clinical College of Xuzhou Medical University, Huaian, China‌‌; NYU Grossman School of Medicine: New York University School of Medicine, UNITED STATES OF AMERICA

## Abstract

To establish a scientifically grounded and feasible intravenous infusion management protocol for patients with Aggressive behavior due to mental disorders. Methods: Guided by Donabedian’s theory, an initial draft of the management protocol was developed through a comprehensive literature review and semi-structured interviews with clinical experts. From June to July 2024, two rounds of expert consultation were conducted, and revisions were made until the final protocol was established. Results: The response rate for both rounds of expert questionnaires was 100%, with authority coefficients of 0.915 and 0.92. After the second round, the coefficients of variation for item importance ranged from 0 to 0.115 and from 0 to 0.107, while Kendall’s W coefficients were 0.506 and 0.628, respectively, indicating statistically significant expert consensus (P < 0.05). Ultimately, the final protocol comprised 3 first-level items, 17 second-level items, and 41 third-level items. Conclusion: The intravenous infusion management protocol developed on the basis of Donabedian’s theory is both rigorous and targeted, providing a foundation for the comprehensive management of intravenous infusion in patients with aggressive behavior.

## 1. Introduction

The prevalence of mental disorders has been steadily increasing, placing significant pressure on health systems and imposing a substantial economic burden on society [1,2]. Aggressive behavior is a common manifestation during the acute phase of mental illness [3,4] typically characterized by extreme anxiety, hyperexcitability, tension, hostility, and physical aggression. Such symptoms not only exacerbate patients’ physical and psychological distress but also increase energy expenditure, thereby elevating the risks of metabolic syndrome and cardiovascular complications, and in severe cases, directly threatening patient safety [5,6]. Intravenous infusion plays a vital role in replenishing nutrition and supporting recovery in agitated patients. However, compared with the general patient population, managing intravenous infusion in these individuals presents unique challenges, including difficulties in catheter insertion, higher risks during various procedural steps [7], and increased psychological stress and workload for caregivers [8]. Existing intravenous infusion management guidelines [9–12] are designed for general use and do not adequately address the specific needs of psychiatric patients with poor treatment adherence or cooperation. Consequently, these guidelines fail to fully meet the therapeutic requirements and quality control standards necessary for this population, highlighting a critical gap in clinical practice. Furthermore, intravenous infusion is a continuous process in which failure at any stage may compromise overall care quality. Donabedian’s theoretical model [13], which evaluates quality from the perspectives of structure, process, and outcome, provides a systematic framework for addressing this challenge. Previous studies applying Donabedian’s framework in nursing have demonstrated its effectiveness in improving both care processes and outcomes [14–16]. However, to date, no infusion management protocol tailored to psychiatric patients with aggressive behavior has been developed using this framework.

Therefore, this study addresses the existing gap by developing a scientifically rigorous, feasible, and comprehensive management protocol for intravenous infusion in patients with aggressive behavior due to mental disorders. This protocol is expected to enhance nursing quality, improve patient safety, and provide a structured approach for clinical practice.

## 2. Materials and Methods

### 2.1. Study design, Setting, and Period

This methodological study was conducted from March to July 2024 in tertiary hospitals in Hebei and Shandong Provinces, China. Guided by Donabedian’s theoretical framework, the study followed a multi-step process: evidence collection through literature review, semi-structured interviews, and two rounds of expert consultation.

### 2.2 Literature review

As the first step of protocol development, a systematic literature review was conducted using the “6S” evidence model [17]. Searches were carried out in Chinese and international databases (CNKI, Wanfang, PubMed, Web of Science, Embase, CINAHL, JBI EBP Database, Cochrane, etc.), guideline repositories (NICE, SIGN, IGIN, RNAO), and professional websites (WHO, BMJ Best Practice, UpToDate). The search period was from database inception to March 2024. Inclusion criteria: (1) studies involving psychiatric patients with aggressive behavior receiving intravenous infusion; (2) management or intervention of infusion care and complication prevention; (3) study designs including RCTs, systematic reviews, guidelines, expert consensus, and clinical practice standards; (4) publications in English or Chinese. Exclusion criteria: conference abstracts, proposals, duplicates, or incomplete data.

From 1,735 initial records, 62 studies were retained after screening, and 14 highly relevant articles were finally included [7–9,18–28]. Quality was appraised using the JBI critical appraisal tool [29]. These findings served as the evidence base for drafting the initial protocol.

### 2.3 Semi-structured interviews

Purposive sampling was used to recruit participants from tertiary hospitals in Hebei and Shandong Provinces. Nursing managers, ward nurses, and patient caregivers with direct experience in intravenous infusion management for psychiatric patients with aggressive behavior were invited through hospital nursing departments and clinical wards. In total, 12 nursing managers, 14 ward nurses, and 12 caregivers participated. Interviews were conducted in a quiet environment, audio-recorded with participants’ permission, and each lasted 20–30 minutes. Data were analyzed using NVivo 11 software following Colaizzi’s 7-step method to extract key themes.

### 2.4 Expert consultation

Fifteen experts from eight tertiary hospitals participated in two rounds of consultation. Experts were eligible if they had ≥10 years of relevant professional experience, held a mid-level professional title or above (associate professor or above for nursing management experts), and possessed at least a bachelor’s degree. Experts who had not been active in the field within the past year were excluded. The same group of experts participated in both rounds. Their research backgrounds included psychiatric nursing management, clinical psychiatric nursing, psychotherapy, and intravenous therapy.

The consultation questionnaire included protocol items rated on a 5-point Likert scale, with additional space for suggestions and supplementary items. Items with mean importance >3.5 and coefficient of variation <0.25 were considered to have reached consensus. For items that did not fully meet the predefined consensus criteria or were considered borderline (e.g., mean importance scores close to the threshold or relatively higher coefficients of variation), expert comments were reviewed in detail. Dissenting opinions and qualitative feedback were systematically summarized and discussed by the research team. Based on these discussions, items were either revised for clarity, merged with conceptually similar items, or retained for reassessment in the subsequent round. Items were not deleted solely based on statistical criteria; instead, both quantitative indicators and expert qualitative judgments were jointly considered to ensure content relevance and clinical applicability.

### 2.5 Statistical analysis

Data were analyzed using SPSS 27.0. Expert authority was quantified using an authority coefficient, and consensus was assessed via mean scores, coefficient of variation, and Kendall’s W. Statistical significance was set at P < 0.05.

### 2.6 Ethical considerations

The study protocol was reviewed and approved by the institutional ethics committee (Approval number: 202316). All participants were fully informed of the study purpose, procedures, potential risks, and their right to withdraw at any time, and written informed consent was obtained prior to data collection. Confidentiality and anonymity were strictly maintained throughout the study.

With respect to the proposed protocol, ethical and legal considerations related to risk management and the use of protective restraint were carefully addressed. Protective restraint is defined as a last-resort measure, to be applied only when less restrictive interventions are insufficient to ensure patient and staff safety. Its use should comply with relevant ethical principles and legal requirements, including clear indications, minimal duration, continuous assessment, and timely discontinuation. The protocol emphasizes respect for patient dignity, proportionality, and minimization of harm, supported by appropriate documentation, staff training, and institutional oversight.

## 3. Results

### 3.1. Results of expert consultation

#### 3.1.1. Expert engagement and authority.

Both rounds of expert questionnaires achieved a 100% response rate. The suggestion rates were 53.33% in the first round and 20% in the second round. The authority coefficients (Cr) were 0.915 and 0.920, both exceeding the 0.80 threshold. (see Table 1).

**Table 1:** Expert engagement and authority (n = 15).

Round	Response rate (%)	Suggestion rate (%)	Authority coefficient (Cr)
1st	100	53.33	0.915
2nd	100	20	0.92

#### 3.1.2. Degree of consensus and coordination.

In the first round, the coefficients of variation (CV) for item importance ranged from 0 to 0.115, and Kendall’s W was 0.506. In the second round, CV values ranged from 0 to 0.107, and Kendall’s W increased to 0.628 (P < 0.001), reflecting improved consensus among experts (see Table 2).

**Table 2:** Degree of consensus and coordination among experts (n = 15).

Round	CV range	Kendall’s W	P
1st	0–0.115	0.506	<0.001
2nd	0–0.107	0.628	<0.001

#### 3.1.3. Expert modification suggestions.

In the first round of consultation, experts provided 32 suggestions. After merging similar comments, 7 items were revised, 1 item was combined, and 8 new items were added. In particular, items with borderline quantitative indicators or divergent expert opinions were refined through wording revisions, item merging, or retention for further evaluation in the second round, based on comprehensive consideration of expert feedback. The main modifications included: (1) expanding the item on infusion safety management to specify operational procedures, emergency response protocols, and annual drills; (2) clarifying ambiguous terms such as “staff education” (revised to “ideological education”), “experienced nurses” (defined as ≥10 years of service) [30], and “puncture method” (revised to “puncture process”); (3) aligning protective restraint nursing with the Expert Consensus on the Implementation and Discontinuation of Protective Restraint; (4) revising “soothing” to “verbal de-escalation and environmental modification techniques”; and (5) standardizing complication management according to the Standards for Intravenous Therapy Nursing Practices. In addition, several new elements were incorporated, such as establishing an emergency call system, specifying training content and assessment mechanisms, including agitation level assessment, detailing measures for peripheral venous protection, adding specialized risk management (e.g., violence, suicide, harm to others), specifying educational content, protecting patient privacy, merging overlapping items, and providing formulas for indicator calculations.

In the second round, further refinements were made. These included specifying a particular risk assessment tool for specialized risk management and reorganizing humanistic care items into pre-infusion, intra-infusion, and post-infusion phases to enhance clarity and practical applicability.

3.2. Intravenous infusion management protocol for psychiatric patients with aggressive behavior (see Table 3)

## 4. Discussion

This study, guided by Donabedian’s framework, developed an intravenous infusion management protocol for psychiatric patients with aggressive behavior through literature review, semi-structured interviews, and Delphi expert consultation. The high response rate, strong authority coefficients, and significant Kendall’s W values indicated reliable consensus. These results are consistent with prior methodological reviews confirming that structured Delphi processes with iterative rounds and transparent consensus indicators enhance scientific rigor [31,32]. Comparable Delphi studies in psychiatric emergencies have also demonstrated that context-specific consensus contributes to developing practical, safety-oriented protocols [33].

**Table 3:** Intravenous Infusion Management Protocol for Psychiatric Patients with Aggressive behavior.

Program Content	Importance Score ($\overset{―}{x}\pm s$)	Coefficient of Variation
1. Structure Level		
1.1 Team Formation	5.000 ± 0.000	0.000
1.1.1 A multidisciplinary team—including clinical nurses, physicians, psychotherapists or consultants, and nutritionists—collaborates in the development and implementation of individualized intravenous therapy protocols [23,24,26,27].	4.267 ± 0.414	0.099
1.1.2 Division of Responsibilities [9,21]:– Clinical nurses: infusion care, risk screening, and health education.– Physicians: management of agitation symptoms, dynamic assessment of infusion outcomes, and treatment adjustment.– Psychotherapists/consultants: assessment of psychological status and provision of counseling.– Nutritionists: nutritional risk assessment and formulation of nutrition plans.	4.267 ± 0.458	0.107
1.1.3 Team Requirements: Enhance communication and promote information sharing among team members.	5.000 ± 0.000	0.000
1.2 System Management	4.933 ± 0.258	0.052
1.2.1 Develop and refine the safety management system, operational procedures, and emergency response protocols for intravenous infusion in psychiatric settings [21,26].	4.867 ± 0.352	0.072
1.3 Personnel Management	4.933 ± 0.258	0.052
1.3.1 Allocate nursing tasks rationally with flexible staffing per shift.	4.933 ± 0.258	0.052
1.4 Environmental Management	4.867 ± 0.352	0.072
1.4.1 Infusion Location: Use designated infusion areas [22] with single rooms for each patient.	5.000 ± 0.000	0.000
1.4.2 Environmental Requirements: Ensure a clean and comfortable environment, minimize exposure to excessive light and noise [8], and ideally provide monitoring devices [22]along with an emergency call system [8].	4.933 ± 0.258	0.052
1.4.3 Environmental Safety: Assign dedicated personnel for management; secure mobile infusion stands; conduct safety checks before and after infusion; and eliminate hazardous items (e.g., sharp objects, movable furniture) from the area, while preventing unauthorized access or crowding around the patient [8].	5.000 ± 0.000	0.000
1.5 Education and Training	5.000 ± 0.000	0.000
1.5.1 Ideological Education: Conduct regular and targeted safety education to reinforce infusion safety awareness.	5.000 ± 0.000	0.000
1.5.2 Training and Assessment: Provide training covering infusion-related theory, core skills, safety management, procedures, monitoring indicators, new techniques, and key aspects of nursing assessment, communication, psychological care, health education, and legal issues; conduct annual theoretical and practical assessments to ensure competency in preventing infusion-related complications [18,19].	4.067 ± 0.258	0.063
1.6 Quality Management	5.000 ± 0.000	0.000
1.6.1 Utilize quality improvement methods (e.g., PDCA cycles, nursing case studies) at least once a year to continuously enhance infusion quality [19,21,22,26].	4.867 ± 0.352	0.072
2. Process Level		
2.1 Infusion Assessment	4.933 ± 0.258	0.052
2.1.1 Pre-infusion Assessment [8,9,28]: A joint evaluation by medical and nursing staff that includes the patient’s age, condition, allergy history, vascular status, limb mobility, intravenous therapy plan, drug properties, puncture site, psychological state, level of cooperation, and the awareness of both patients and family members regarding intravenous infusion; In addition, the assessment should cover the risk of violence (using the BVC scale), suicide risk (using the NGASR), and the degree of agitation (using scales such as PANSS-EC, BARS, OASS, or MOAS).	5.000 ± 0.000	0.000
2.1.2 Intra-infusion Dynamic Assessment: Monitor the puncture site, infusion rate, adverse reactions, as well as the patient’s psychological status, degree of agitation, and psychiatric symptoms.	5.000 ± 0.000	0.000
2.1.3 Post-infusion Assessment: Reassess the puncture site and psychiatric symptoms to prepare for subsequent care.	4.133 ± 0.351	0.085
2.2 Symptom Management	4.867 ± 0.352	0.072
2.2.1 Non-pharmacological Control: Preferentially employ verbal de-escalation and environmental modification techniques [7], with medical protective restraint as a last resort [8,22].	5.000 ± 0.000	0.000
2.2.2 Pharmacological Control: When non-pharmacological methods are insufficient, consider pharmacological interventions in consultation with physicians and per medical orders [8].	4.067 ± 0.258	0.063
2.3 Occupational Safety	4.867 ± 0.352	0.072
2.3.1 Identity Verification: Strictly enforce patient identification protocols using at least two verification methods [21,28].	4.933 ± 0.258	0.052
2.4 Puncture Management	4.867 ± 0.352	0.072
2.4.1 Puncture Personnel: Nurses with at least 10 years of experience, extensive puncture expertise, and established patient trust.	4.933 ± 0.258	0.052
2.4.2 Puncture Equipment: Preferentially use intravenous indwelling needles.	5.000 ± 0.000	0.000
2.4.3 Prioritize forearm puncture sites to facilitate infusion completion and minimize dislodgement or embolism risk in agitated patients [25,27].	5.000 ± 0.000	0.000
2.4.4 Puncture Process: Use a two-nurse approach, with one providing psychological support and the other performing the puncture.	4.933 ± 0.258	0.052
2.4.5 Fixation Method: Implement appropriate peripheral venous protection measures (e.g., using transparent dressings) based on the patient’s condition [19].	5.000 ± 0.000	0.000
2.5 Risk Management	4.933 ± 0.258	0.052
2.5.1 Complication Management: Monitor for infusion reactions (e.g., fever, circulatory overload, phlebitis, air embolism) and device-related complications (e.g., drug extravasation, nerve injury), following the ‘Standards for Intravenous Therapy Nursing Practices’.	5.000 ± 0.000	0.000
2.5.2 Specialized Risk Management: Implement protocols specific to psychiatric care to prevent self-harm, suicide, and violent behavior.	5.000 ± 0.000	0.000
2.5.3 Perform 15–30-minute rounds to monitor patient status and infusion-site conditions, ensure timely management of adverse reactions, and maintain infusion safety [9,28].	5.000 ± 0.000	0.000
2.5.4 Protective Restraint Nursing: For patients undergoing protective restraint during infusion, implement care in accordance with the “Expert Consensus on the Implementation and Discontinuation of Protective Restraint,” minimizing restraint while ensuring safety [8].	5.000 ± 0.000	0.000
2.6 Health Education	4.933 ± 0.258	0.052
2.6.1 Target Audience: Patients and caregivers [21]. Prior to education, assess their needs and comprehension capacity to implement individualized health education.	5.000 ± 0.000	0.000
2.6.2 Educational Content: Should include, but not be limited to, disease knowledge, the purpose and significance of infusion management, and information on managing agitation—coupled with periodic evaluations [21,25,28].	4.133 ± 0.352	0.085
2.6.3 Educational Methods: Employ methods such as face-to-face individual or group sessions.	4.200 ± 0.414	0.099
2.6.4 Frequency of Education: For patients and families who are uncooperative or lack understanding, increase the frequency of education to ensure continuous learning.	4.133 ± 0.352	0.085
2.7 Humanistic Care	4.933 ± 0.258	0.052
2.7.1 Create a comfortable infusion environment by maintaining a clean, tidy room and providing items such as cups and call bells.	5.000 ± 0.000	0.000
2.7.2 Select puncture sites considering vascular status and lifestyle habits, prioritizing the non-dominant arm, avoiding lower limb veins, and using behavioral interventions to relieve pain. [23,25].	5.000 ± 0.000	0.000
2.7.3 Employ gentle techniques during care and use encouraging, instructive language during rounds to express understanding of the patient’s feelings; use non-verbal communication when appropriate.	4.933 ± 0.258	0.052
2.7.4 Consider scheduling infusions during the daytime to promote better sleep and overall health [18].	4.133 ± 0.352	0.085
2.7.5 Protect and respect patient privacy by considering their beliefs and customs; when exposure is necessary, use appropriate coverings.	5.000 ± 0.000	0.000
2.7.6 Provide effective psychological care by employing empathy, suggestion, and distraction techniques; actively address reasonable patient needs and respond promptly to improve cooperation.	4.867 ± 0.352	0.072
2.8 Auxiliary Equipment	4.867 ± 0.352	0.072
2.8.1 Pre-infusion: Based on assessment results, consider using vascular visualization technologies (e.g., infrared, transillumination) [24,27]. For patients at risk of self-harm, suicide, or violence, use needle-free connectors to prevent injury [25].	4.933 ± 0.258	0.052
2.8.2 Intra-infusion: Utilize devices such as flow control systems [9,25] and infusion bottle alarm systems; select appropriate physical fixation devices (e.g., protective shields or gloves) [25].	4.067 ± 0.258	0.063
2.9 Line Maintenance	4.933 ± 0.258	0.052
2.9.1 Assess the catheter insertion site and remove the catheter early when indicated; in severely agitated patients, remove it immediately after infusion [21,28].	4.867 ± 0.352	0.072
3. Outcome Level		
3.1 Nursing Outcomes	4.933 ± 0.258	0.052
3.1.1 Focus on infusion quality indicators [9], including infusion-related adverse events or complications (events per 1,000 catheter-days), patient satisfaction (assessed using standardized surveys), and unplanned catheter removal rates (number of unplanned removals per total catheter-days).	4.800 ± 0.414	0.086
3.2 Occupational Protection	4.867 ± 0.352	0.072
3.2.1 Monitor the incidence of needle-stick injuries (number of injuries per infusion episode × 100%).	5.000 ± 0.000	0.000

Compared with existing infusion guidelines designed for general patient populations [7,34], our protocol responds to the unique needs of psychiatric patients, who often show poor cooperation and heightened risks. The incorporation of emergency call systems, risk management strategies, and detailed competency criteria mirrors findings from European Delphi consensus guidelines on suicidal crisis management, which emphasized structured risk assessment and phased care strategies [35]. By aligning structural, process, and outcome dimensions, our protocol ensures both technical precision and humanistic care, echoing evidence that Donabedian’s triad provides a robust model for nursing quality improvement [36].

From an implementation perspective, the proposed protocol is intended to be integrated into routine psychiatric nursing workflows rather than applied as an additional standalone intervention. The structure–process–outcome framework enables nursing managers to translate the protocol into standard operating procedures, role-based task allocation, and quality monitoring indicators that align with existing ward management systems. In clinical practice, the protocol can be operationalised through a phased implementation approach, beginning with core safety-related components such as risk assessment, environmental management, and staff training, followed by the gradual incorporation of multidisciplinary collaboration and outcome evaluation. Importantly, the protocol was designed with flexibility to accommodate resource-limited psychiatric wards. While multidisciplinary participation and advanced equipment are recommended at the structural level, the protocol prioritises essential nursing actions that can be implemented using existing personnel and basic infrastructure. Key elements, including non-pharmacological de-escalation strategies, standardized assessment tools, and environmental safety measures, can be applied without substantial additional resources. This adaptable design enhances the feasibility of implementation across psychiatric settings with varying levels of staffing and resource availability.

The expert-driven refinements incorporated during the consultation process—such as specifying standardized risk assessment tools, reorganizing humanistic care items according to infusion phases, and standardizing complication management—further support the clinical applicability of the protocol [37]. These features are consistent with existing evidence suggesting that structured training and clearly defined operational standards can enhance nurse confidence and patient safety in psychiatric settings. Nevertheless, several limitations should be acknowledged. First, the expert panel was drawn from tertiary hospitals in two provinces, which may limit the generalizability of the findings to other regions or levels of care. Second, although a high level of consensus was achieved, the protocol has not yet undergone empirical validation. Future research should therefore focus on stepwise empirical evaluation of the protocol. Initial feasibility studies and pilot implementations in psychiatric wards are warranted to assess practicality, staff adherence, and acceptability in routine clinical practice. Building on these findings, implementation research and outcome evaluations could further examine the protocol’s effectiveness in improving patient safety, reducing staff burden, and optimizing clinical outcomes. Relevant outcome measures may include infusion-related adverse events, unplanned catheter removal, treatment continuity, patient satisfaction, as well as indicators of nursing workload, occupational stress, and confidence in managing aggressive behavior. In addition, the transferability of the proposed protocol to other regions or international contexts may be influenced by cultural, organisational, and health-system factors. Variations in perceptions of aggressive behavior, ward management structures, staffing patterns, and resource availability across psychiatric settings may affect implementation of specific components. Nevertheless, the core principles of the protocol—namely structured risk assessment, process-oriented infusion management, and outcome-based quality monitoring—are grounded in the Donabedian framework and are broadly applicable. Context-specific adaptation is therefore recommended to ensure alignment with local clinical practices and health-system characteristics.

## 5. Conclusion

Based on Donabedian’s theory, this study has developed an intravenous infusion management protocol for psychiatric patients with Aggressive behavior that encompasses first-, second-, and third-level items. Covering the entire process of intravenous infusion management, the protocol is scientifically sound, reliable, targeted, and feasible—and it may serve as a valuable reference for clinical nursing practice in psychiatric settings. Although this study did not empirically validate the protocol, it provides a theoretically grounded and consensus-based framework for clinical application. Future research will focus on feasibility testing and pilot implementation in psychiatric wards, particularly to evaluate practicality, staff adherence, and preliminary clinical outcomes prior to large-scale application.

## Supporting information

S1 File Supplementary record file. (RAR)
